# Characteristics of Developmental and Healing Process of Docetaxel-Induced Lower Limb Edema in Patients with Stage IV Breast Cancer: A Case Series

**DOI:** 10.1089/pmr.2022.0059

**Published:** 2023-02-20

**Authors:** Yuko Imakata, Junko Sugama, Sayumi Ichihashi, Fumiya Oohashi, Masato Kobayashi, Makoto Oe

**Affiliations:** ^1^ Division of Health Sciences, Graduate School of Medical Sciences, Kanazawa University, Ishikawa, Japan.; ^2^ Department of Adult Nursing, Ishikawa Prefectural Nursing University, Ishikawa, Japan.; ^3^ Research Center for Implementation Nursing Science Initiative, Fujita Health University, Aichi, Japan.; ^4^ Department of Clinical Nursing, Faculty of Health Sciences, Institute of Medical, Pharmaceutical and Health Sciences, Kanazawa University, Ishikawa, Japan.; ^5^ Department of Radiological Technology, Faculty of Health Sciences, Institute of Medical, Pharmaceutical and Health Sciences, Kanazawa University, Ishikawa, Japan.

**Keywords:** chemotherapy, compression therapy, docetaxel, initial finding, severe edema, ultrasonography

## Abstract

**Background:**

Management of docetaxel-induced edema is important as severe edema may lead to discontinuation of chemotherapy. Patients with stage IV breast cancer (BC) treated with docetaxel have shown lower limb edema; however, details of its developmental and healing processes are unknown, and thus management strategies have not been established. The aim of this study was to investigate the characteristics of the development and healing process of docetaxel-induced lower limb edema in stage IV BC patients.

**Methods:**

This prospective observational study was conducted on patients with BC who were administered docetaxel between September 2020 and September 2021 at a National Hospital in Japan. Skin changes such as pitting test, circumference, along with ultrasound images and subjective symptom changes were evaluated. The progression of these changes was compared between patients with stage IV and non-stage IV disease.

**Results:**

Five patients were enrolled in the study, of which two and one patients with stage IV and non-stage IV disease, respectively, developed lower limb edema. Early signs of lower limb edema were observed in ultrasound images, 15 cm below the peroneal head, before edema was confirmed by the pitting test and subjective symptoms. In patients with stage IV disease, edema worsened to Grade 3, and reduced four months after the end of drug administration.

**Conclusion:**

For patients with stage IV disease, care should be initiated from the time the early signs are observed using ultrasound and continued for up to four months after the end of docetaxel administration.

## Introduction

Docetaxel is one of the most commonly used chemotherapeutic agents for patients with breast cancer (BC). Edema is a known characteristic side effect of docetaxel,^1^ which occurs due to increased vascular permeability and protein leakage outside the blood vessels.^2^ Edema has been reported in about 30% of the patients who received docetaxel therapy.^3^ The management of docetaxel-induced edema is of paramount importance, since severe edema would result in discontinuation of chemotherapy, which would be detrimental to the patient.^4^

However, in BC patients, focus is on surgical lymphadenectomy and radiotherapy-induced lymphedema, not on docetaxel-induced edema. Moreover, there are guidelines for lymphedema.^5^ But lymphedema guidelines have also been applied to docetaxel-induced edema, which has a different mechanism. Moreover, although the actual situation of lymphedema in BC patients has been reported,^6^ the actual situation of docetaxel-induced edema has hardly been reported.

To establish the guidelines for docetaxel-induced edema management, it is necessary to correctly diagnose the entity, especially edema in lower limbs that is not affected by lymph node dissection and radiotherapy. There are few reports of docetaxel-induced lower limb edema in BC patients. It has been reported that docetaxel-induced lower limb edema returned to the baseline before taxane administration six months later.^7^ And we found that the factor of lower extremity edema is stage IV disease.^8^

Monitoring skin changes in the lower limbs, including dermis and subcutaneous tissue, is required to establish the diagnosis of docetaxel-induced lower limb edema in stage IV BC patients. The diagnoses of chronic edema and lymphedema using ultrasonic diagnostic equipment have been reported; however, the diagnostic method of docetaxel-induced lower limb edema has not been clarified.^6,9,10^

Therefore, this study aimed to elucidate the characteristics of skin changes and subjective symptoms in stage IV BC patients who received docetaxel therapy, which would contribute to the establishment of management strategies for docetaxel-induced lower limb edema.

## Methods

### Study population

BC patients, aged ≥20 years, who were administered docetaxel therapy between September 2020 and September 2021 at a National Hospital in Japan formed the study population for this prospective observational study. The patients were monitored from the start of outpatient docetaxel administration to one month after the cessation of treatment. If lower limb edema was observed in the patients, they were followed up until the edema resolved or up to six months from the beginning of treatment. Patients with comorbidities such as severe cardiac/hepatic/renal dysfunction and patients with deep vein thrombosis were not included in the study. At the facility, if the edema worsened or if the patient requested, it was recommended that the patient be instructed to raise the lower extremities and wear stockings.

### Data collection

Parameters such as age, sex, body mass index, activities of daily living, medical history, chemotherapy regimen, total docetaxel dosage (mg),^11^ BC stage,^8^ hemoglobin level, and malnutrition status were retrieved from the medical records to elucidate patient characteristics.

The regulations for edema limbs from the Common Terminology Criteria for Adverse Events (CTCAE) were used to classify edema (Grade 1/Grade 2/Grade 3).^12^ Pitting test was done according to the modified Fukazawa method, to evaluate the degree of edema (0/1/2/3/nonpitting edema).^13^ For pitting test and observation of the circumference: (1) the circumference passing through the distal side of the first to fifth metatarsal bones; (2) the circumference of the ankle joint; (3) 5 cm peripheral from the patellar joint.^5^

Portable echo Sonosite iViz (FUJIFILM, Tokyo, Japan) was used to obtain the ultrasonographic images. A linear probe (L38v) with frequency band of 5 to 10 MHz was used 15 cm below the right fibula head.^9^ The right lower limb was used for circumference measurement, pitting test, and ultrasonography.

These parameters were recorded on an everyday basis between 10:00 am and 12:00 pm, with patients sitting in a chair with their legs extended.

Patients were interviewed about five symptoms, including dullness of sensation, tingling, weakness of lower limbs, weight, and pain, to elucidate the subjective symptoms of edema.^14^

As part of the institutional chemotherapy side effect management, chemotherapy-certified nurses observed the patients for symptoms of all possible side effects of chemotherapy and educated them on how to deal with any side effects.

### Ethical considerations

This study was conducted in accordance with the principles of the Declaration of Helsinki. The study was approved by the Ethics Committee of the University and the research facility (75-1, R02-041).

### Analysis

Changes in the skin and subjective symptoms were analyzed on a case-by-case basis. Post which, the process of these changes was compared between stage IV and non-stage IV participants. The ultrasound images of the lower limbs were qualitatively described by the researchers. The researchers who did the data collection were certified nurse specialists in cancer and were supervised by specialists in wound nursing research and clinical radiology.

## Case Descriptions

A total of five individuals participated in this study, of whom two were diagnosed with stage IV BC, one with stage III, and the remaining two with stage II. Lower limb edema was observed in two patients with stage IV and one with stage II cancer (Table 1). None of the patients had their dose of docetaxel reduced due to symptoms of edema.

**Table 1.** Characteristics of Lower Limb Edema in Patients with Stage IV and Non-Stage IV Breast Cancer

	Stage IV patients			Non-stage IV patients	
Stage	Stage IV	Stage IV	Stage II	Stage III	Stage II
Case	A	B	C	D	E
Age	70s	60s	70s	70s	70s
Sex	Female	Female	Female	Female	Female
BMI	21.9	31.6	30.4	15.2	20.0
ADL	Wheelchair	Independence	Independence	Independence	Independence
Disease	Subarachnoid hemorrhage Anxiety	Diabetes Systemic lupus erythematosus	Endometrial cancer Thyroid cancer	Hyperlipidemia Osteoporosis	Atrial fibrillation
Purpose of chemotherapy	Recurrence Metastasis	Recurrence Metastasis	Postoperative	Preoperative	Preoperative
Chemotherapy regimens	Docetaxel, Trastuzumab, Perjeta	Docetaxel, Trastuzumab, Perjeta	Docetaxel, Cyclophosphamide	Docetaxel, Trastuzumab, Perjeta	Docetaxel, Trastuzumab, Perjeta
Hb (g/dL)	7.4	9.8	12.6	11.4	10.0
TP (g/dL)	5.0	6.1	7.0	6.1	6.7
Total dose of docetaxel (mg)	600	660	440	400	400
Edema	+	+	+	−	−

+, presence of edema; −, absence of edema.

ADL, activities of daily living; BMI, body mass index; Hb, hemoglobin; TP, total protein.

### Case description of lower limb edema development in a stage IV BC participant

**FIG. 1.**
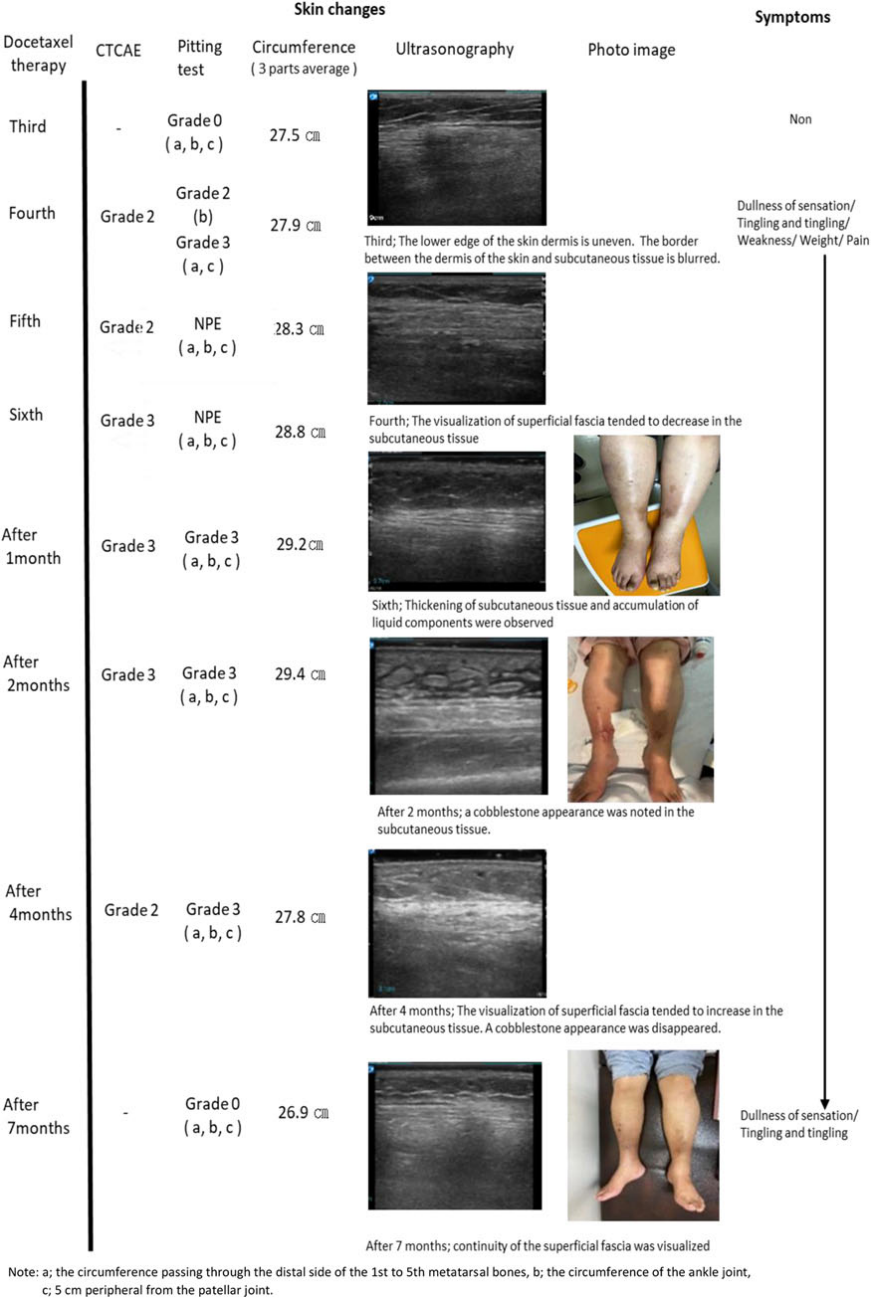
Representative case description of docetaxel-induced lower limb edema in Stage IV breast cancer. CTCAE, Common Terminology Criteria for Adverse Events; NPE, nonpitting edema.

A 60-year-old female participant with stage IV BC enrolled for this study (Fig. 1). Grade 2 lower limb edema appeared after the administration of the fourth dose. Edema worsened to Grade 3 by the time the sixth dose was administered. The condition improved to Grade 2 four months after the end of administration and resolved completely after seven months.

Circumference of the lower limb returned to the initial state after seven months.

Ultrasonography initially showed an uneven lower edge of skin dermis. The border between dermis and subcutaneous tissue blurred by the administration of third dose of docetaxel. By the fourth administration, the superficial fascia became invisible. Thickening of subcutaneous tissue and accumulation of liquid components were observed by the sixth round of drug administration. Two months after the end of drug administration, a cobblestone appearance was noted in the subcutaneous tissue, which disappeared after four months. Seven months after the end of drug administration, the superficial fascia was flat in the subcutaneous tissue.

Subjective symptoms of edema appeared by the fourth round of docetaxel administration, and some symptoms persisted even after seven months, when edema had disappeared.

### Comparison between the lower limb edema development in stage IV and non-stage IV BC patients

**FIG. 2.**
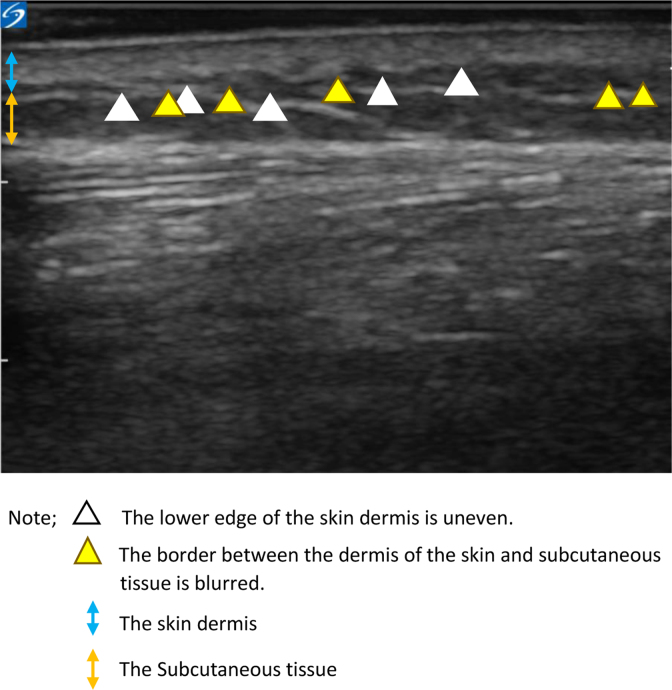
Initial findings of lower limb edema with docetaxel of ultrasonic images of 15 cm below the fibula head.

In both stage IV and non-stage IV BC patients, CTCAE and pitting test showed that lower limb edema developed after the administration of the fourth dose of docetaxel (Table 2). In addition, the pitting test confirmed that ultrasonographic findings such as uneven lower edge of skin dermis and blurring of the border between the dermis and subcutaneous tissue appears before edema in both stage IV and non-stage IV BC patients (Fig. 2).

**Table 2.** Comparison Between the Lower Limb Edema Development in Stage IV and Non-Stage IV Breast Cancer Patients

	Stage IV patients	Non-stage IV patients	
Stage	Stage IV (*n* = 2)	Stage II (*n* = 1)	Stage II (*n = 1) Stage III (n* = 1)
Edema	Edema	Edema	No edema
CTCAE v5.0	1. At the fourth dose, edema appeared. 2. At the sixth dose or after one month, edema worsened to Grade 3. 3. After four months, edema was reduced to Grade 2 or Grade 1.	1. At the fourth dose, edema appeared. 2. After two months, edema worsened to Grade 2. 3. After four months, edema disappeared.	—
Pitting test (average of 3 sites)	1. At the fourth dose, edema appeared. 2. After one month, edema worsened to Grade 3.	1. At the fourth dose, edema appeared. 2. After one month, edema worsened to Grade 2.	—
Circumference measurement (average of 3 sites)	1. After one month, maximum value attained. 2. After seven months, returned to size before the appearance of edema.	1. After two months, maximum value attained. 2. After four months, returned to size before the appearance of edema.	—
Ultrasound images	1. At the third dose, the lower edge of the skin dermis was uneven. The border between the dermis of the skin and subcutaneous tissue was blurred. 2. At the fourth dose, superficial fascia became invisible. 3. After one to two months, cobblestone appearance was seen	1. At the second dose, the lower edge of the skin dermis was uneven. The border between the dermis of the skin and subcutaneous tissue was blurred. 2. At the fourth, the superficial fascia became invisible.	1. From the second dose, the lower edge of the skin dermis was flat. The superficial fascia was flat in subcutaneous tissue.
Subjective symptoms	1. At the fourth dose, dullness of sensation, tingling, weakness of lower limbs, weight gain, and pain appeared. 2. After seven months, dullness of sensation and tingling remained.	1. At the third dose, dullness of sensation, weakness of lower limbs, and weight gain. 2. After four months, dullness of sensation remained.	1. At the second or third dose, dullness of sensation, weakness of lower limbs, and pain appeared. 2. After one month, symptoms were resolved (*n* = 1). After five months, dullness of sensation and weakness of lower limbs remained (*n* = 1).

CTCAE = Common Terminology Criteria for Adverse Events.

However, in stage IV BC patients, lower limb edema was characterized by the appearance of severe Grade 3 edema and cobblestone appearance in ultrasound images of the subcutaneous tissue. Moreover, in stage IV patients, the circumference of lower limbs recovered after seven months after drug administration, compared with faster recovery in non-stage IV patients.

**FIG. 3.**
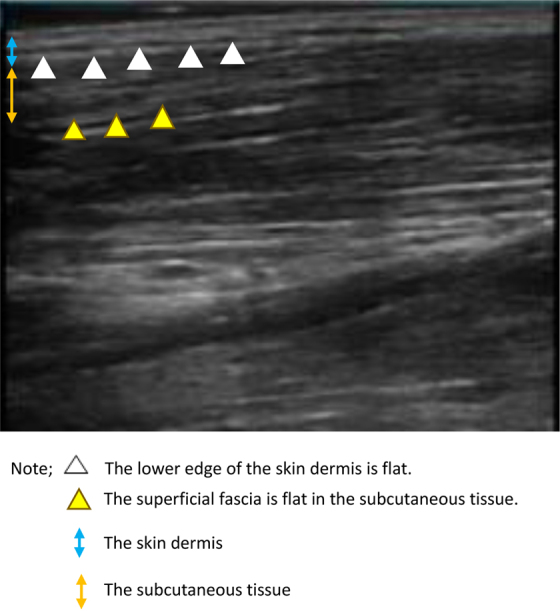
Findings in the lower limbs of patients who did not develop edema of ultrasonic images of 15 cm below the fibula head.

In non-stage IV BC patients who did not have edema, ultrasound images showed a flat lower edge of the skin dermis. Moreover, the superficial fascia was flat in the subcutaneous tissue (Fig. 3).

## Discussion

To the best of our knowledge, this is the first prospective study to report the characteristics of lower limb edema in stage IV BC patients who received docetaxel therapy. We found that early signs of lower limb edema were observed in ultrasound images, even before edema could be elicited by the pitting test. Moreover, our study showed that lower limb edema in stage IV BC patients was more serious than in non-stage IV patients.

For stage IV patients, appropriate management of edema from the time of early signs by ultrasound imaging may improve the patients' quality of life during and after docetaxel treatment. Our findings may be important in designing the protocols for the management and prevention of exacerbation of lower limb edema in patients with BC undergoing docetaxel therapy.

### Early signs of lower limb edema

Early ultrasonographic signs of lower limb edema were uneven lower edge of the skin dermis and blurring of the border between dermis and subcutaneous tissue.

Edema due to docetaxel occurs due to increased vascular permeability.^4^ The papillary layer of the dermis is rich in capillaries and is said to be most susceptible to edema when inflammation occurs.^15^ Administration of docetaxel increases the capillary permeability in the papillary layer, causing the liquid component to leak into the interstitial tissue. It is suggested that edema is first observed in the dermis of the skin. Therefore, it is recommended that edema be managed right from the time these early symptoms appear, to improve the patient's quality of life during and after docetaxel therapy.

### Severity of lower limb edema and duration of care

Lower limb edema in stage IV BC patients was more serious, and took a long time to resolve completely. Furthermore, the subcutaneous tissue showed a cobblestone appearance by ultrasonography. Stage IV BC patients use docetaxel more frequently than non-stage IV patients. Stage IV BC patients have histological destruction due to metastasis. Previously, stage IV BC was found to be a risk factor for occurrence of lower limb edema^8^; however, this study suggests that it also affects the severity. Moreover, lower limb edema decreased in all three subjects, four months after the end of drug administration. Regarding the time of relief, there is only a single report of improvement before six months after administration.^7^ Therefore, care is recommended up to four months after the end of docetaxel administration.

### Course of edema symptoms and application of compression therapy

Subjective symptoms appeared before the onset of edema, regardless of the presence or absence of edema, and persisted even after the end of docetaxel administration. We believe that these subjective symptoms may be a combination of symptoms due to edema, and peripheral neuropathy as a side effect of docetaxel. Therefore, it is difficult to distinguish between the early findings of edema and subjective symptoms, which is why it is important to perform image evaluation using ultrasonography.

Continuity loss of the superficial fascia seen in the ultrasound image at the fourth dose administration, in which dents were observed, is similar to echo findings in lower limb lymphedema.^9^ Edema caused by docetaxel may progress to systemic edema and get complicated by heart failure.^16^ Compression therapy is recommended for lymphedema, but is contraindicated in heart failure.^17^ In contrast, in the care of venous ulcers of the lower extremities, there is limited consensus regarding the use of compression therapy due to the risk of concomitant heart failure.^18^ Based on these findings, it is recommended to treat docetaxel-induced edema with low-pressure compression therapy, while paying close attention to signs of heart failure.

This study had some limitations. First, the depth of the ultrasound imaging device was limited to the subcutaneous tissue. Second, in our institution, the first dose of docetaxel was administered in an inpatient setting. However, the study was conducted at the time when docetaxel therapy was initiated on an outpatient basis. Therefore, the occurrence and process of edema cannot be compared with those before the start of docetaxel therapy, and limits the accuracy of assessing the cure of edema.

## Conclusions

This study revealed that lower limb edema was characterized as severe in patients with stage IV BC. Furthermore, it was found that early signs of lower limb edema were observed in ultrasound images even before edema could be elicited by the pitting test. For stage IV patients, we recommend that care be initiated from the time the early signs are observed on ultrasound images, and this should be continued for up to four months after the end of docetaxel administration. Studies should be conducted in future to clarify the effect of compression therapy in the prevention of severe docetaxel-induced edema.

## Abbreviations Used

ADL: activities of daily living
BC: breast cancer
BMI: body mass index
CTCAE: Common Terminology Criteria for Adverse Events
Hb: hemoglobin
NPE: nonpitting edema
TP: total protein
